# Clinical Improvement of Disseminated Acanthamoeba Infection in a Patient with Advanced HIV Using a Non-Miltefosine-Based Treatment Regimen in a Low-Resource Setting

**DOI:** 10.3390/tropicalmed7020024

**Published:** 2022-02-04

**Authors:** Denasha L. Reddy, Eunice van den Berg, Wayne Grayson, Matilda Mphahlele, John Frean

**Affiliations:** 1 Division of Infectious Diseases, Department of Internal Medicine, Chris Hani Baragwanath Academic Hospital, University of the Witwatersrand, Johannesburg 2193, South Africa; 2 Department of Anatomical Pathology, Faculty of Health Sciences, University of the Witwatersrand, Johannesburg 2193, South Africa; Eunice.vandberg@nhls.ac.za (E.v.d.B.); graysonw@ampath.co.za (W.G.); 3 National Health Laboratory Service, Chris Hani Baragwanath Academic Hospital, Johannesburg 1804, South Africa; 4 Ampath Laboratories, Johannesburg 2055, South Africa; 5 Department of Dermatology, Chris Hani Baragwanath Academic Hospital, University of the Witwatersrand, Johannesburg 2193, South Africa; 0602022k@students.wits.ac.za; 6 National Institute for Communicable Diseases, National Health Laboratory Service, Johannesburg 2192, South Africa; johnf@nicd.ac.za; 7 Wits Research Institute for Malaria, University of the Witwatersrand, Johannesburg 2193, South Africa

**Keywords:** acanthamoeba, miltefosine, HIV, opportunistic infections, amphotericin B deoxycholate

## Abstract

Disseminated *Acanthamoeba* species infection is likely an underrecognized and underdiagnosed opportunistic infection in patients with advanced human immunodeficiency virus (HIV) disease in South Africa. It presents a unique clinical challenge in that the diagnosis can be difficult to establish and management options are limited in low-resource settings. To our knowledge, there is a paucity of literature to date on the successful use of combination treatment options for patients in low-resource settings without access to miltefosine. We present a case describing the clinical improvement of disseminated *Acanthamoeba* infection in a patient with advanced HIV using a non-miltefosine-based treatment regimen. The case serves to highlight that *Acanthamoeba* sp. infection should be considered as a differential diagnosis for nodular and ulcerative cutaneous lesions in patients with advanced HIV in South Africa, and that although there are alternative options for combination treatment in countries without access to miltefosine, efforts should be made to advocate for better access to miltefosine for the treatment of acanthamoebiasis in South Africa.

## 1. Introduction

*Acanthamoeba* species are classified as free-living protozoa, as they are widely distributed in nature (soil, air, sewage, seawater, tap and bottled water) in both feeding trophozoite and dormant cystic forms, and do not depend on a host for transmission and spread [1,2]. Several species of bacteria may be harboured intracellularly within clinical and environmental isolates of *Acanthamoeba* spp., allowing for endosymbiosis as the intracellular bacteria are protected from adverse conditions, and can evade host defenses [1,2,3,4]. *Acanthamoeba* spp. can cause opportunistic infections, such as granulomatous amoebic encephalitis (GAE), nasopharyngeal, cutaneous and systemic infections in immunocompromised people, or non-opportunistic infections such as amoebic keratitis in otherwise healthy people [1,2,3,4]. Systemic *Acanthamoeba* spp. infections usually have a slow, subclinical course, compared with the acute disease of amoebic keratitis [1]. The portal of entry of the organism in systemic infections is usually through breaches in the skin or through the respiratory tract, eventually spreading to the brain haematogenously [1,2,3,4]. The gold standard for diagnosis is isolation, cultivation and subsequent identification of *Acanthamoeba* spp. from tissue, although molecular techniques such as polymerase chain reaction (PCR) and 18s rDNA-sequencing provide greater sensitivity [1,2,3,4]. 

Disseminated and cutaneous *Acanthamoeba* spp. infections have been recognized as opportunistic infections in patients with advanced HIV, following the first reported case in 1986 [3]. Most reported cases were diagnosed post-mortem, as the diagnosis was often missed and many patients had a fulminant clinical course [3]. In addition to GAE, other manifestations of *Acanthamoeba* spp. infections in this group were chronic sinusitis, otitis, leukocytoclastic vasculitis, amoebic osteomyelitis and cutaneous lesions in the form of sinuses and skin ulcers [3]. Although *Acanthamoeba* species are ubiquitous, they remain an uncommon and possibly underdiagnosed cause of infection [1,2,3,4]. The distribution of *Acanthamoeba* spp. in South Africa has been described in zoological and environmental sampling studies [5,6,7], but the prevalence of opportunistic infections due to *Acanthamoeba* spp. in South Africa is largely unknown, as the only published clinical reports describe cases of amoebic keratitis [8,9].

## 2. Case Report

The patient was a 42-year-old former security guard from Soweto, Johannesburg, with known HIV infection for the past 14 years, on standard first-line antiretroviral treatment for adults (tenofovir, emtricitabine and efavirenz) according to the national antiretroviral treatment guideline at the time of presentation [10]. He received his antiretroviral therapy at a local clinic, and admitted to two previous episodes of defaulting therapy. He had been admitted to hospital twice in the preceding two years for the management of opportunistic infections: the first admission was for pulmonary tuberculosis, and the second was for community-acquired pneumonia. He presented to our tertiary academic hospital with a 4-month history of headaches, reduced visual acuity in the right eye and malaise, and a 2-month history of numerous discrete skin lesions distributed over his limbs, face and abdomen, sparing the palms, soles and mucous membranes. The first lesion appeared on the lateral aspect of his foot, then spread to involve his legs, arms, chest and abdomen. The skin lesions were tender but not pruritic. The patient also complained of arthralgia involving his wrists, ankles and knees, which resulted in reduced mobility. There was no associated fever, cough or diarrhoea. He did not wear contact lenses and there was no history of trauma to either eye. He had worked previously as a security guard, but had been unemployed for the past two years, following his diagnosis of pulmonary tuberculosis. He was a widower and lived with his 13-year-old daughter in an informal settlement in Soweto.

**Figure 1:**
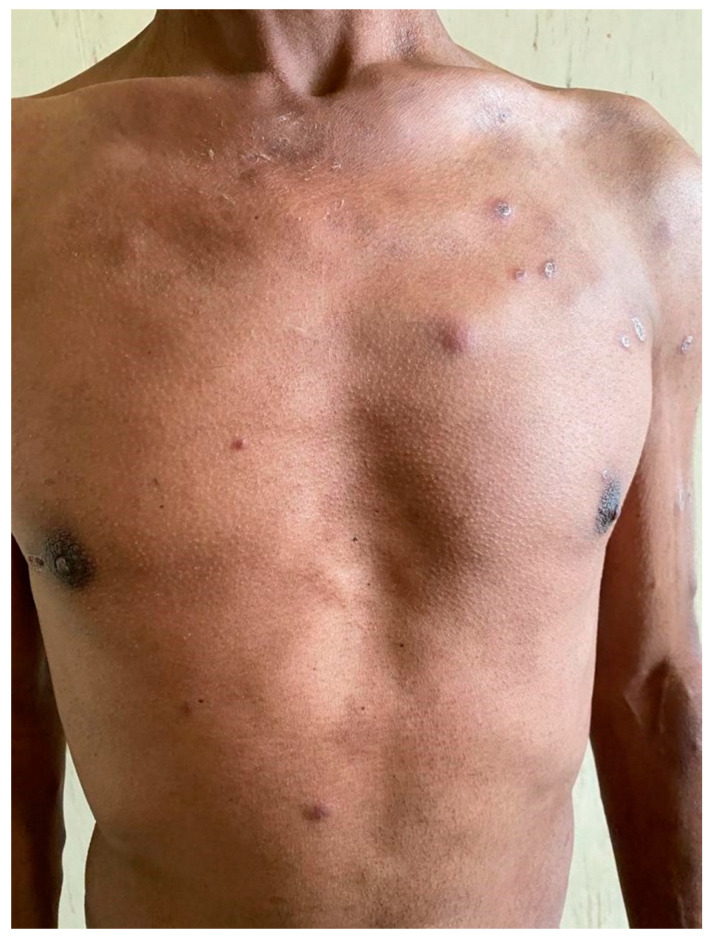
Patient’s torso with lesions at different stages: skin-coloured papules, subcutaneous nodules and plaques with a necrotic centre.

**Figure 2:**
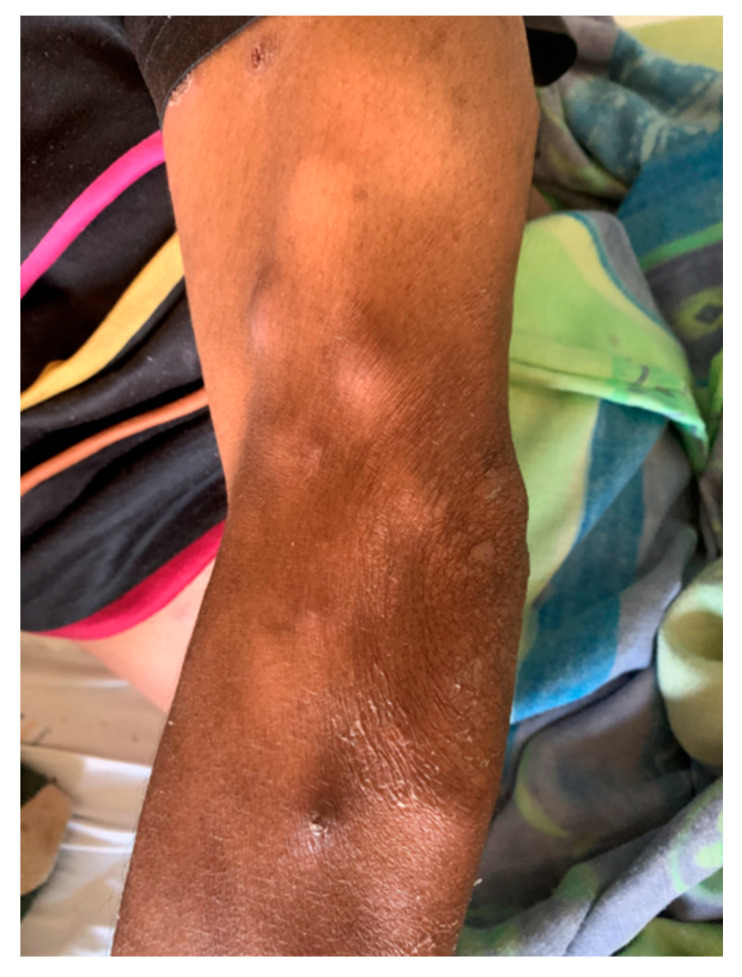
Patient’s left upper arm and elbow, showing dermal nodules.

**Figure 3:**
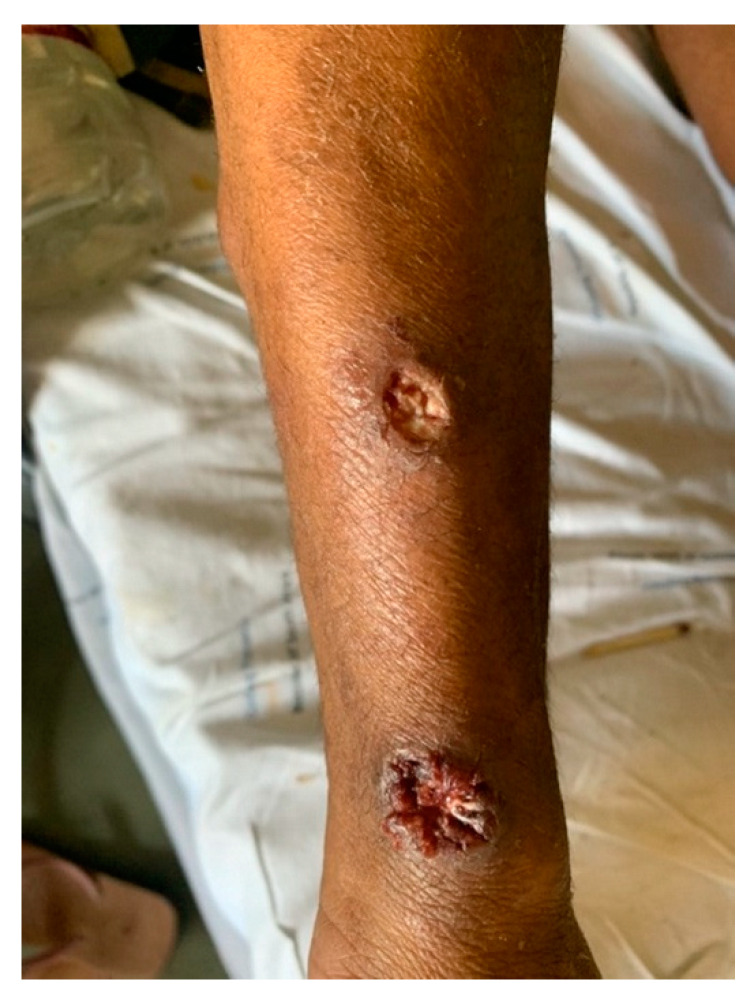
Patient’s right forearm, showing skin ulcers originating from dermal nodules.

**Figure 4:**
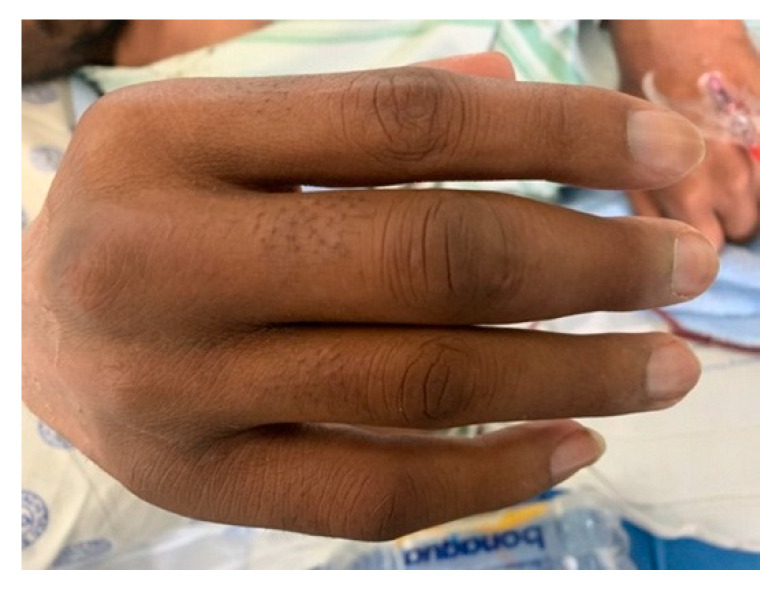
Patient’s right hand on admission, showing joint and finger swelling.

On initial clinical examination, the patient appeared wasted and chronically ill. He had generalized shotty lymphadenopathy and numerous subcutaneous nodules involving his anterior chest (Figure 1), arms (Figure 2 and Figure 3) and lower limbs. The lesions were noted to begin as papules, progress to nodules and then spontaneously ulcerate and form scabs. There was synovitis of the small joints in his right hand, as well as dactylitis (Figure 4). The cardiorespiratory, gastrointestinal and neurological examinations were all unremarkable. Of note, there was no evidence of a chronic meningitis or any focal neurological deficits. Formal ophthalmic assessment revealed an old inactive uveitic process affecting the right eye, with no evidence of keratitis in either eye. Initial dermatological clinical assessment included a differential diagnosis of lymphoma, deep fungal infection and syphilis.

**Figure 5:**
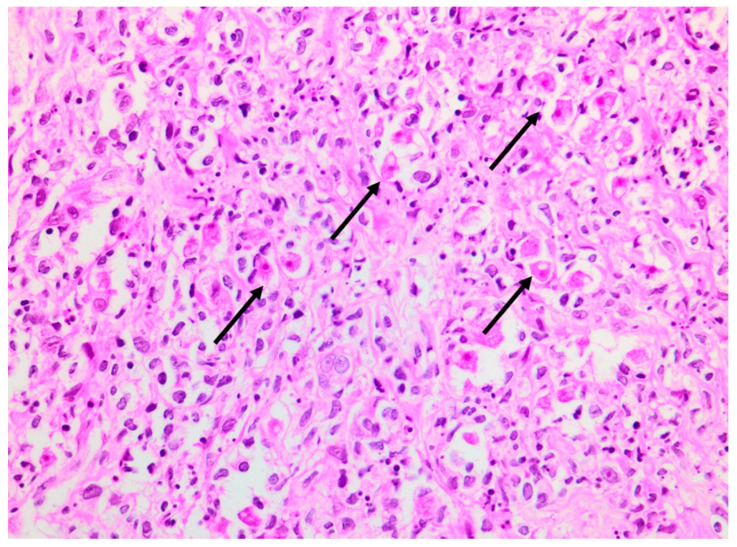
Photomicrograph of skin punch biopsy showing numerous amoebic organisms (arrows) and a polymorphous background inflammatory infiltrate (haematoxylin and eosin, ×400).

**Figure 6:**
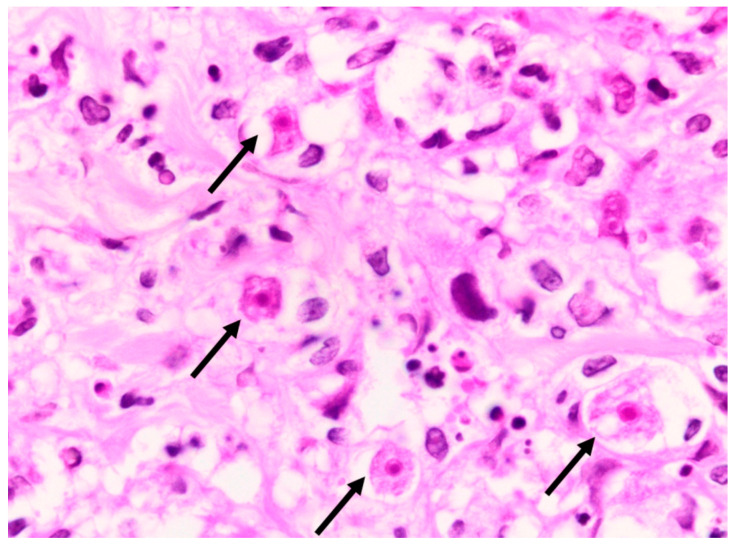
*Acanthamoeba* organisms (arrows), as seen under oil immersion. Note the characteristic targetoid appearance of the nuclei (haematoxylin and eosin, ×1000).

A deep skin punch biopsy was obtained from one of the lesions on the forearm, and submitted in 10% buffered formalin. The haematoxylin and eosin-stained sections revealed a polymorphous nodular deep dermal and subcutaneous inflammatory cell infiltrate comprising neutrophils, histiocytes, lymphocytes and eosinophils in response to conspicuous numbers of free-living amoebic organisms (Figure 5). Each round trophozoite was seen to possess a characteristic targetoid eosinophilic nucleus (Figure 6). Only rare, encysted forms were noted in the inflamed areas. Occasional subcutaneous vessels showed vasculitis. Erythrophagocytosis was conspicuously absent. There was no preponderance of histiocytic giant cells. The periodic acid-Schiff (PAS) stain was positive in some of the organisms, but the Grocott methenamine silver, TriPAS and Ziehl-Neelsen stains were negative. A diagnosis of cutaneous acanthamoebiasis was rendered, and the infectious diseases service was consulted. Further skin biopsies were sent to the national reference parasitology laboratory at the National Institute for Communicable Diseases (NICD). Cultures and PCR testing of the isolate performed at the NICD according to published methods [8,9] confirmed the presence of an *Acanthamoeba* species, with further 18s rRNA gene-sequencing identifying the isolate as a T4 strain, *Acanthamoeba polyphaga* (>99% identity with GenBank sequence accession no. MN153018.1).

**Figure 7:**
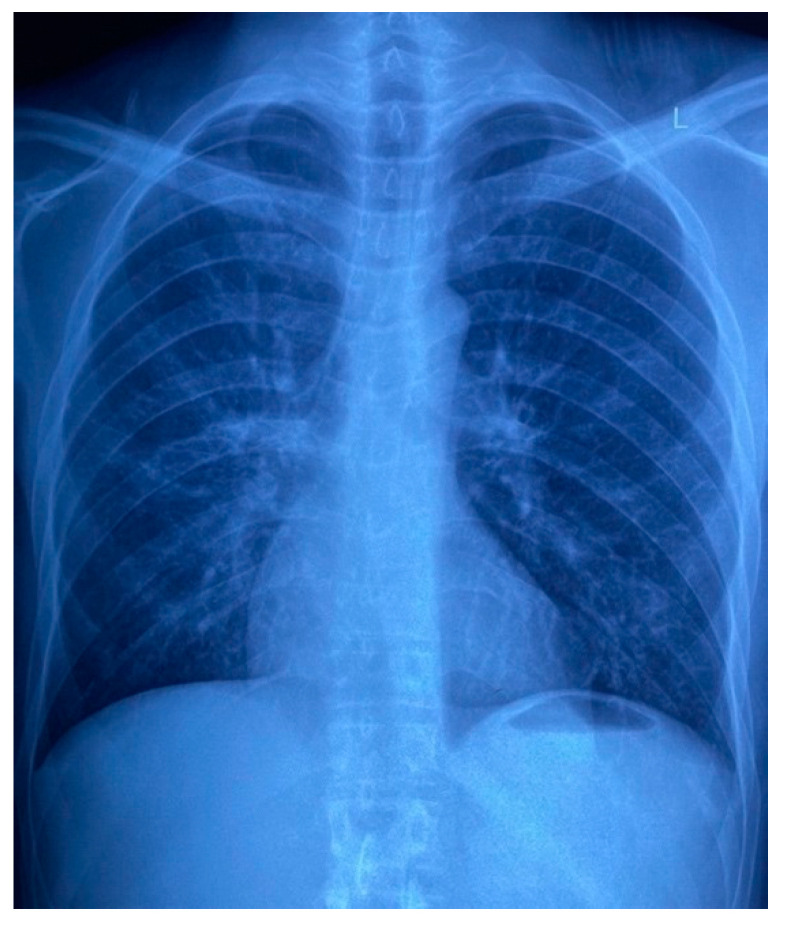
Patient’s chest X-ray on admission, demonstrating a nodular infiltrate in the lower lobe and lingula of the left lung.

**Figure 8:**
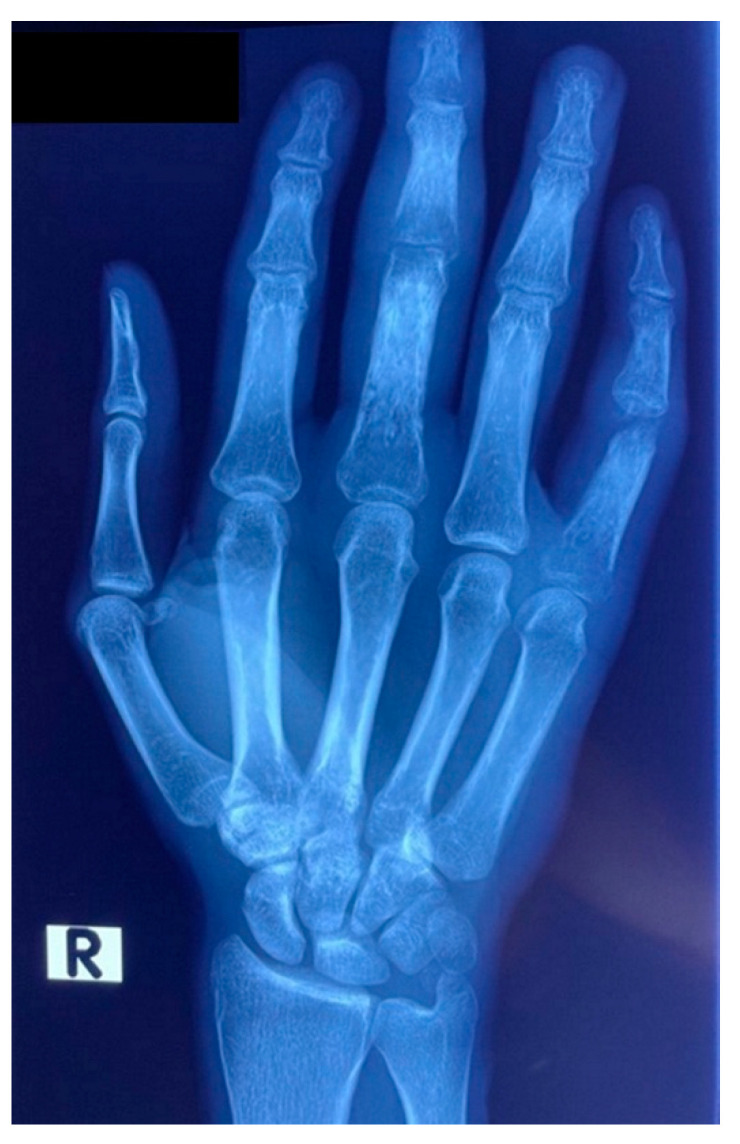
X-ray of patient’s right hand, demonstrating a periosteal reaction in the 3rd proximal phalanx and osteolysis of the 5th proximal interphalangeal joint.

Routine laboratory results on admission showed a normocytic anaemia, lymphopenia, a raised C-reactive protein of 141 mg/L and a normal procalcitonin of 0.09 μg/L. The CD4 cell count was 5 cells/μL and an HIV viral load was 110,000 copies/mL, confirming immunological and virological treatment failure, respectively. Reflex serum cryptococcal antigen screening and a *Treponema pallidum* antibody test were negative. Contrast-enhanced CT and MRI brain imaging, and cerebrospinal fluid examination did not reveal any abnormalities. A bone marrow aspirate and trephine biopsy excluded an infiltrate, and the anaemia was thought to be secondary to HIV infection. Of note, the admission chest X-ray revealed a nodular infiltrate in the lower lobe and lingula of the left lung (Figure 7) and the X-ray of the patient’s right hand demonstrated a periosteal reaction in the third proximal phalanx and osteolysis of the fifth proximal interphalangeal joint (Figure 8), which suggested a systemic process with multiorgan involvement.

In order to confirm disseminated *Acanthamoeba* sp. infection involving the lung, a bronchoscopy for bronchoalveolar lavage and biopsy was ordered, but subsequently cancelled, due to the clinical deterioration of the patient. A decision was made to commence treatment for disseminated acanthamoebiasis without further delay. After the review of recommended treatment regimens in the literature and advice from experts, we attempted to source miltefosine, without success. A modified combination regimen was initiated and continued for the next six weeks: amphotericin B deoxycholate (1 mg/kg intravenously daily), fluconazole (400 mg orally 12-hourly), mebendazole (100 mg orally 12-hourly), cotrimoxazole (10 mg/kg/day orally divided 12-hourly), azithromycin (500 mg orally daily) and metronidazole (400 mg orally 8-hourly). Incidentally, a mycobacterial blood culture performed on admission became positive after 15 days of incubation and acid-fast bacilli (AFBs) were observed, necessitating the addition of antituberculous treatment (rifampicin, isoniazid, ethambutol and pyrazinamide) empirically. The mycobacterial isolate was later identified as *Mycobacterium intracellulare*. Although voriconazole was recommended for its amoebicidal activity, fluconazole was used instead in order to avoid drug interactions, as the patient was also on rifampicin for the mycobacterial infection. Mebendazole was used instead of albendazole, due to unavailability of the latter.

**Figure 9:**
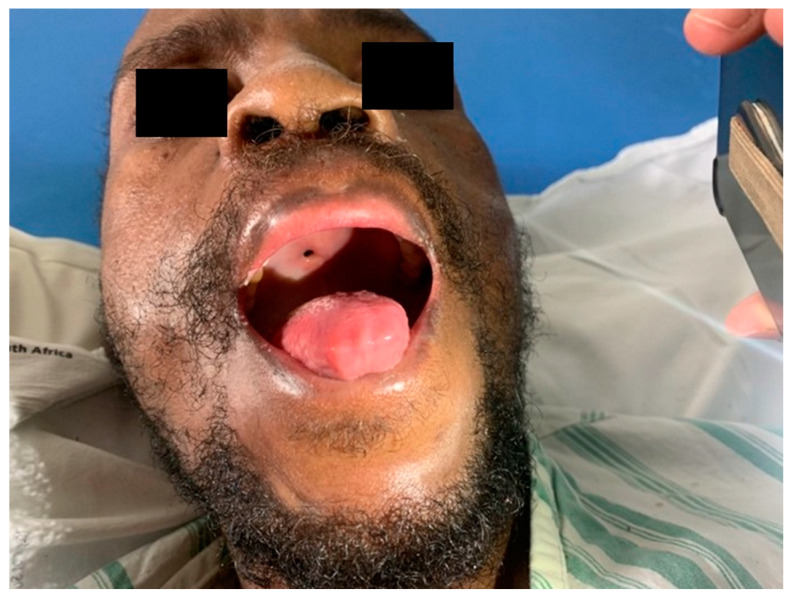
2 mm diameter fistula in hard palate.

There was noticeable improvement in the size of the skin lesions and inflammation of the right hand after one week of combination therapy. Unfortunately, follow up photographs and histological examination of the skin lesions that demonstrated remission and the absence of amoebae were not performed. The patient was switched to second-line antiretroviral treatment (zidovudine, lamivudine and lopinavir/ritonavir) after three weeks of treatment [10]. During the course of treatment for disseminated acanthamoebiasis, the patient developed a 2 mm diameter fistula on his hard palate, communicating between his nasal space and oral cavity (Figure 9). The hard palate biopsy revealed no infiltrate, and only superficial fibrinopurulent exudate. Bacterial culture showed a moderate growth of *Escherichia coli* and *Enterococcus faecalis,* thought to be contaminants, although co-infection with *Acanthamoeba* sp. was possible.

Despite developing a systemic bacterial healthcare-associated infection (HAI) and temporary pancytopenia (thought to be amphotericin B deoxycholate-related), the patient had a good clinical response to six weeks of inpatient treatment for disseminated acanthamoebiasis and mycobacterial infection, and was to be discharged home for outpatient follow-up at the infectious disease clinic on an oral regimen of fluconazole, mebendazole, cotrimoxazole and azithromycin (to be continued for a minimum of six months). Unfortunately, he remained in hospital for a further three weeks as he required placement in a care facility, due to poor social circumstances and lack of family support. He developed another bacterial HAI whilst awaiting placement and demised, almost three months after admission. The HAI was diagnosed clinically by the treatment team on the basis of a rise in inflammatory markers, new pyrexia and infected buttock pressure ulcers which were the most likely the infective source. Laboratory results showed a new pancytopenia, a raised C-reactive protein of 123 mg/L, and a raised procalcitonin of 18.46 μg/L. Unfortunately, no organism was cultured from the blood culture specimens and a definitive pathogen was not identified. No academic post-mortem was performed as no consent could be obtained from the next of kin.

## 3. Discussion

The case serves to highlight that *Acanthamoeba* sp. infection should be considered as a differential diagnosis for nodular and ulcerative cutaneous lesions in patients with advanced HIV in South Africa, and that early diagnosis and combination treatment can result in clinical improvement. A case of *Balamuthia mandrillaris* infection in South Africa has been diagnosed recently (manuscript submitted for publication) and consequently *B. mandrillaris* infection must be considered an important differential diagnosis of cutaneous acanthamoebiasis. Important differences that distinguish *Acanthamoeba* spp. and *Balamuthia* spp. infections include the following: *Acanthamoeba* spp. are more likely to act opportunistically and infect immunocompromised patients, whereas *Balamuthia* spp. occurs more commonly in immunocompetent patients; the cutaneous lesions in acanthamoebiasis tend to be nodules with frequent ulceration, usually distributed on the extremities and face, while in *Balamuthia* spp., the primary lesion is usually a centrofacial non-ulcerated plaque; finally, the histopathological examination of infected tissues reveals abundant trophozoites in *Acanthamoeba* spp. infection, but multinucleated giant cells, frequent angiocentric inflammation, and sparse trophozoites in *Balamuthia* spp. infection [1,2,3,11].

There are no specific preventive measures for disseminated *Acanthamoeba* spp. infection in immunocompromised patients, although reversal of the immunosuppression remains a key component of prevention and management in this group [1,2,3,4].

Disseminated *Acanthamoeba* sp. infection is difficult to treat, as there is no single drug that is effective and no standardized treatment regimen, due to the relative paucity of cases, lack of reproducible data, and lack of randomized control trials [1,12]. Combination regimens are empirical, but have proven useful, as many drugs exhibit amoebostatic but not amoebicidal activity [3,12]. Many other factors also affect patient outcomes: time to initiation of treatment, inoculum of amoebae, virulence and antimicrobial susceptibility of the organism, and immune status of the host [1,12].

The literature describes cases of GAE and cutaneous *Acanthamoeba* spp. infection without neurological involvement that were successfully treated with combination regimens of pentamidine isethionate, sulfadiazine, 5-fluorocytosine, fluconazole or itraconazole, although these treatments were often discontinued due to drug toxicities [2,12]. For cutaneous infections, topical applications of chlorhexidine gluconate and ketoconazole cream were added [2,12]. Other drugs with in vitro amoebostatic activity against *Acanthamoeba* spp. that were used as part of this patient’s treatment regimen were cotrimoxazole, azithromycin and amphotericin B [3]. Miltefosine has been described as having amoebicidal activity, and also penetrates the blood–brain barrier [4,12]. Recent case reports provide anecdotal evidence that miltefosine can be used successfully in combination regimens to treat disseminated *Acanthamoeba* spp. infections in immunocompromised patients, and that its use has shown a survival advantage in some cases [13,14,15]. Current expert advice suggests using a combination regimen for disseminated acanthamoebiasis of pentamidine, fluconazole, and miltefosine, and the optional addition of cotrimoxazole, metronidazole and azithromycin [16]. Amphotericin B has not been shown to inhibit the growth of acanthamoebae in vitro, although there is anecdotal evidence that it can result in cure when used as part of a combination regimen [17].

A review of the most recent case reports describing regimens for the treatment of cutaneous acanthamoebiasis used in patients with HIV revealed the use of a wide range of therapeutic combinations with inconsistent outcomes [18,19,20]. A case series by Galarza et al. describing cutaneous acanthamoebiasis infection in immunocompetent and immunocompromised patients showed that three out of the five patients in the series were HIV-infected [18]. These patients received a combination treatment regimen of itraconazole and amphotericin B, with a good clinical response, although two of the three patients subsequently developed other opportunistic infections and died [18]. Nachega et al. described a case of aggressive rhinosinusitis, secondary to *Acanthamoeba* infection, in a patient with advanced HIV (CD4 cell count of 100 cells/μL) that was successfully treated with high-dose amphotericin B and 5-fluorocytosine, in addition to surgical debridement of the affected sinuses [19]. This case report included a review of 16 other documented cases of *Acanthamoeba* rhinosinusitis in HIV-infected patients, demonstrating a high mortality (13 out of 16 patients) in this subset of patients who had received varying combination treatment regimens, of which the most frequent was an azole (ketoconazole or itraconazole) and 5-fluorocytosine [19]. The three survivors in this review had all received a combination of itraconazole and 5-fluorocytosine, with additions of amphotericin B and rifampicin in the first patient, amphotericin B in the second patient, and rifampicin in the third patient [19]. Another case report by Paltiel et al. described a good clinical response to the treatment of disseminated cutaneous acanthamoebiasis in a patient with advanced HIV (CD4 cell count of 3 cells/μL) using a combination of intravenous pentamidine, oral 5-fluorocytosine and sulfadiazine [20]. Paltiel et al. included a review of the response to common antimicrobials (5-fluorocytosine, pentamidine, itraconazole, ketoconazole, fluconazole, amphotericin B, azithromycin and metronidazole) used to treat immunocompromised patients with disseminated acanthamoebiasis without CNS involvement, which showed no obvious advantage to the use of any single antimicrobial [20].

In view of the patient’s deteriorating clinical condition, likely disseminated disease, and our inability to source miltefosine, we opted for an aggressive treatment regimen using a combination of the six drugs we had access to: amphotericin B deoxycholate, fluconazole, mebendazole, cotrimoxazole, azithromycin, and metronidazole. The main limitation of this case report was that the authors could not conclusively demonstrate successful treatment of the *Acanthamoeba* infection as a repeat skin biopsy to demonstrate clearance of the pathogen was not undertaken. However, the reduction in the size of the cutaneous lesions and improvement in the patient’s general condition to the point that he was ready for discharge, and a planned outpatient follow-up, did provide circumstantial evidence of clinical improvement, and at the time, another skin biopsy was not thought to be necessary. Unfortunately, images demonstrating the observed clinical improvement are not available, and thus not included in this case report. Another limitation of this case report is that disseminated *Acanthamoeba* infection could not conclusively be excluded as the cause of the patient’s death, as a post-mortem could not be performed. However, the treatment received by our patient has been documented to be successful in some cases of cutaneous acanthamoebiasis in patients with HIV. Furthermore, the diagnosis of an HAI as the cause of death was supported indirectly by clinical and laboratory evidence, as detailed in the case report, and the growth of organisms from the blood culture specimens submitted may have been inhibited in vitro by the broad-spectrum drugs such as cotrimoxazole and azithromycin.

In terms of a way forward, a database for all *Acanthamoeba* spp. infections and other free-living amoebae is needed in South Africa, in order to better describe the prevalence of disease, genotyping of isolates, clinical presentation, and response to treatment. A high index of clinical suspicion and a lower threshold for skin biopsies in immunocompromised patients is warranted, as the diagnosis is likely to be missed. Although there are alternative options for combination treatment in countries without access to miltefosine, efforts should be made to advocate for better access to miltefosine for the treatment of acanthamoebiasis in South Africa, as observational evidence has demonstrated better outcomes when miltefosine is used as part of a combination regimen.

## 4. Conclusions

Our case demonstrates that disseminated *Acanthamoeba* infection in an immunocompromised patient can respond positively to a multidrug treatment regimen that does not include miltefosine, an agent that is not available or affordable in many countries. Better access to miltefosine will benefit patients with the uncommon but frequently fatal systemic infections caused by free-living amoebae.

## Data Availability

No new data were created or analyzed in this study. Data sharing is not applicable to this article.
